# Optimizing respiratory management in resource-limited settings

**DOI:** 10.1097/MCC.0000000000000568

**Published:** 2018-12-13

**Authors:** Rebecca Inglis, Emmanuel Ayebale, Marcus J. Schultz

**Affiliations:** a Lao-Oxford-Mahosot Hospital-Wellcome Trust Research Unit (LOMWRU), Mahosot Hospital, Vientiane, Lao People's Democratic Republic; b Department of Intensive Care, Oxford University Hospital Trust, Oxford, UK; c Department of Anaesthesia, Makerere University, Kampala, Uganda; d Department of Intensive Care, Royal Liverpool and Broadgreen University Hospitals NHS Trust, Liverpool, UK; e Department of Intensive Care; f Laboratory of Experimental Intensive Care and Anesthesiology (LEICA), Amsterdam University Medical Center, University of Amsterdam, Amsterdam, The Netherlands; g Mahidol Oxford Tropical Medicine Research Unit (MORU), Mahidol University, Bangkok, Thailand

**Keywords:** acute respiratory failure, low- and middle-income countries, mechanical ventilation, resource-limited settings, ventilatory support

## Abstract

**Purpose of review:**

This review focuses on the emerging body of literature regarding the management of acute respiratory failure in low- and middle-income countries (LMICs). The aim is to abstract management principles that are of relevance across a variety of settings where resources are severely limited.

**Recent findings:**

Mechanical ventilation is an expensive intervention associated with considerable mortality and a high rate of iatrogenic complications in many LMICs. Recent case series report crude mortality rates for ventilated patients of between 36 and 72%. Measures to avert the need for invasive mechanical ventilation in LMICs are showing promise: bubble continuous positive airway pressure has been demonstrated to decrease mortality in children with acute respiratory failure and trials suggest that noninvasive ventilation can be conducted safely in settings where resources are low.

**Summary:**

The management of patients with acute respiratory failure in LMICs should focus on avoiding intubation where possible, improving the safety of mechanical ventilation and expediting weaning. Future directions should involve the development and trialing of robust and context-appropriate respiratory support technology.

## INTRODUCTION

Since 1990, nearly all countries in the world have experienced an improvement in healthcare access and quality [1]. Nonetheless huge discrepancies remain, with data suggesting that a young adult with a lower respiratory tract infection is still over six times more likely to die in a low-income country than in a high-income country (HIC) [1].

To narrow the gap in health outcomes, there is a need to focus on improving the quality of the care being delivered, especially in low- and middle-income countries (LMICs) [2^▪▪^]. This is unlikely to be achieved by efforts to export an exact replica of the healthcare systems and processes from HICs. Instead it needs the development of bespoke, context-appropriate solutions that are founded on a locally relevant evidence base and are devised and championed by local healthcare professionals.

The current review focuses on the nascent body of literature that pertains to the management of acute respiratory failure and other pathologies requiring respiratory support in LMICs. The aim is to abstract management principles that are of relevance to all settings where resources are scarce, whilst acknowledging the fallacy of overgeneralizing across hugely diverse geographies. 

**Box 1:**
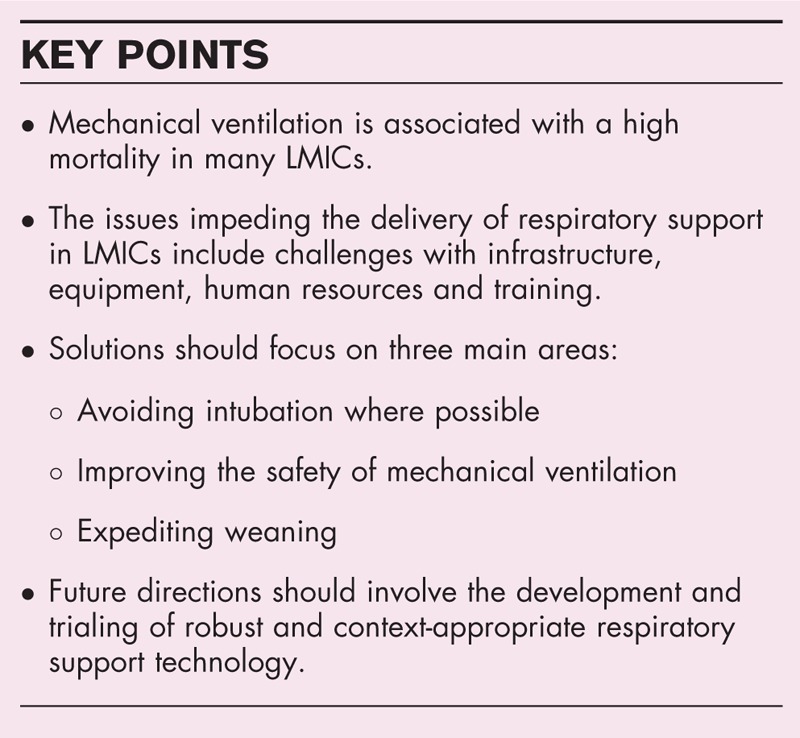
no caption available

## EPIDEMIOLOGY

Our understanding of the epidemiology of acute respiratory failure in LMICs is limited. It is likely to vary considerably across the broad range of country incomes included in the LMIC category. The majority of published data hails from a non-representative selection of large, urban centers, often with a university affiliation, rather than provincial or district level facilities where the bulk of clinical care is conducted [3^▪▪^]. Furthermore, the lowest income countries are consistently underrepresented or absent altogether.

Despite these limitations, a fairly consistent pattern that emerges is that invasive mechanical ventilation in LMICs is associated with high crude mortality, with case series reporting ICU mortality rates of between 36 and 72% [4–7]. In HICs reported mortality rates are much lower, between 32 and 34% [8,9]. There is insufficient information available to closely interrogate the reasons underlying these differences, but they are borne out by the findings from the LUNG SAFE study, a prospective cohort of patients with acute respiratory distress syndrome (ARDS) [10^▪^]. The dataset included 546 patients from 120 ICUs in middle-income countries and demonstrated a strong association between a country's economic status and ARDS survival. This link is starkly illustrated by their finding that for every additional $1000 increase in a country's gross domestic product (GDP) per capita, the odds ratio for hospital mortality in patients with ARDS is 0.983 [10^▪^]. This would imply that an ARDS patient admitted to an ICU in the country with the highest GDP per capita in their study ($81 000) has 0.256 the odds of dying compared with a patient with ARDS admitted to an ICU in the country with the lowest ($1600).

With regard to the spectrum of disease leading to respiratory failure, much of it echoes the case mix in HICs. Diagnoses seen more frequently in LMICs compared with HICs are organophosphate poisoning, obstetric emergencies, envenomation, tetanus and other tropical infections [5,6,11–13]. A common trend across many LMICs is the rise in patients requiring ventilatory support due to trauma. This tallies with the increasing global burden of trauma which disproportionately affects LMICs, where 90% of all road traffic fatalities happen [14], and where three times more cases of traumatic brain injury occur compared with HICs, taking into account relative population sizes [15].

Ventilated patients in LMICs have been shown to be at greater risk of certain ventilator-associated complications than their HIC counterparts. A prime example is ventilator-associated pneumonia (VAP), as evidenced by a systematic review and meta-analysis in Asia [16^▪▪^]. The findings are a substantial cause for concern: not only was the rate of VAP higher, but many of the causative organisms were also highly antibiotic resistant. Previous studies have shown that VAP is associated with increased mortality in LMICs; a prospective multicenter study in Vietnam showed that ventilator-associated infections are also associated with increased patient costs and length of stay [17^▪^].

## THE CHALLENGES

There are several obstacles to delivering high-quality care to patients with acute respiratory failure in resource-limited settings. Although these vary between institutions, they predominantly involve equipment, infrastructure and human resources.

An observational study in India that included 4038 patients from 120 ICUs showed that inadequately equipped ICUs were associated with a higher mortality, even when adjusting for illness severity [11]. Table 1 lists the equipment and infrastructure challenges commonly encountered when providing respiratory support in resource-limited settings, as described in the literature.

**Table 1:** Common equipment and infrastructure challenges

Mechanical ventilators
Absence of training or technical support to operate the ventilator – *manuals rarely provided in the local language*.
No biomedical engineers to maintain and repair equipment – *many machines are sold to LMICs without a maintenance contract.*
Multiple brands and models in use within a single ICU – *due to donations or uncoordinated procurement.*
Frequent need to reuse single-use components, especially ventilator tubing – *disposable tubing is less robust and hard to clean thoroughly.*
Poor access to consumables – *including heat and moisture exchangers and suction catheters, meaning they are reused or omitted.*
Poor access to spare ventilator parts – *flow meters break frequently and leave users unable to monitor the tidal volumes delivered.*
Unreliable oxygen supply of variable quality.
Inconsistent electricity – *voltage variability shortens the lifespan of equipment and mandates the use of a voltage stabilizer, while a back-up generator is needed for power cuts.*
Many ventilators require a compressed air source to run.
Circuit humidification is challenging – *circuit blockages are common, often due to dried secretions in the endotracheal tube (affecting 38% of patients in one case series, some repeatedly)* [13].

Ancillary equipment and investigations
Patient monitoring – *pulse-oximetry access improving but capnography is rarely available.*
Arterial blood gas analysis – *often unavailable or unaffordable leaving no way to monitor ventilation adequacy.*
Few drug pumps for delivering continuous sedation infusions – *many units are dependent on intermittent bolus doses of benzodiazepines making light sedation hard to achieve.*
Lack of on-ventilator imaging – *many units lack portable X-ray equipment or bedside ultrasound.*
Absent/poor quality/fake drugs – *especially opiates, neuromuscular blockers and antibiotics.*

LMIC, low- and middle-income country. Adapted from [18,19^▪^,^20^,^21^,^22^^▪▪^,^23^].

The two main challenges pertaining to human resources are understaffing and inadequate training. In a survey of 13 clinicians from 11 LMICs, a lack of appropriately trained ICU staff was cited as the single greatest barrier to improving the quality of care for critically ill patients [24].

Given this widely held perception, it was surprising that the LUNG SAFE study showed no independent association between ARDS mortality and the ratio of nurses or doctors to ICU beds [10^▪^]. This may simply reflect the fact that this study collected only daytime and not nighttime staffing levels, when staffing shortages are often greatest. Alternatively it could reflect unmeasured variation in the training and efficacy of the healthcare staff.

A recent study in Hong Kong demonstrated a threshold effect, above which increased staffing no longer improves outcomes, rather than a linear association [25^▪^]. The study also showed that as little as one day of inadequate nurse staffing during a patient's ICU admission was associated with an increased risk of death. This reinforces the principle that a continuous minimum staffing presence is necessary to avoid adverse patient outcomes [26], and is an important consideration when it comes to safely implementing mechanical ventilation. It is not sufficient to have good staffing for a portion of the time in the hope that it will compensate for periods of understaffing; constant surveillance is necessary to ensure patient safety.

The other key staffing deficit afflicting many under-resourced ICUs is a lack of physiotherapy capability. As part of the ‘Intensive Care Over Nations’ audit, data were collected on 3713 patients admitted to 299 ICUs in LMICs. In 44% of centers, patients had no regular access to a physiotherapist [27]. Many patients in LMICs thereby suffer the dual jeopardy of insufficient rehabilitation during their inpatient stay combined with fewer community-based services to support them to resume an acceptable quality of life after discharge.

## THE APPROACH TO THE PATIENT WITH RESPIRATORY FAILURE IN A RESOURCE-LIMITED SETTING

Given that the mortality associated with mechanical ventilation is substantially higher in resource-limited settings, it follows that every effort should be made to avoid intubation whenever possible. For those patients where it is unavoidable, the priority should be to liberate them from the ventilator at the earliest opportunity.

The strategy has additional benefits in a context where providing mechanical ventilation to a patient has the potential to occasion catastrophic expenditure in as little as one day. In China, one day on ICU receiving mechanical ventilation in 2017 cost $1212 [28]. The GDP per capita in China in the same year was $8827, meaning that just one day of mechanical ventilation cost more than a month's income.

The exact threshold for intubation – judging when the benefits outweigh the risks – will vary across settings. Local outcome data should inform the discussion with the patient and family to set realistic expectations of duration, cost and prognosis. Regular reevaluation can mitigate the risk of continuing beyond the point of futility, at ongoing daily cost to the family. The World Federation of Societies of Intensive and Critical Care Medicine has drawn up guidance to support local decision-making and triage decisions that emphasizes the importance of avoiding ICU admission in cases where there is little realistic prospect of reversibility or based purely on a patient's ability to pay; the same would apply for mechanical ventilation [29^▪^].

**FIGURE 1:**
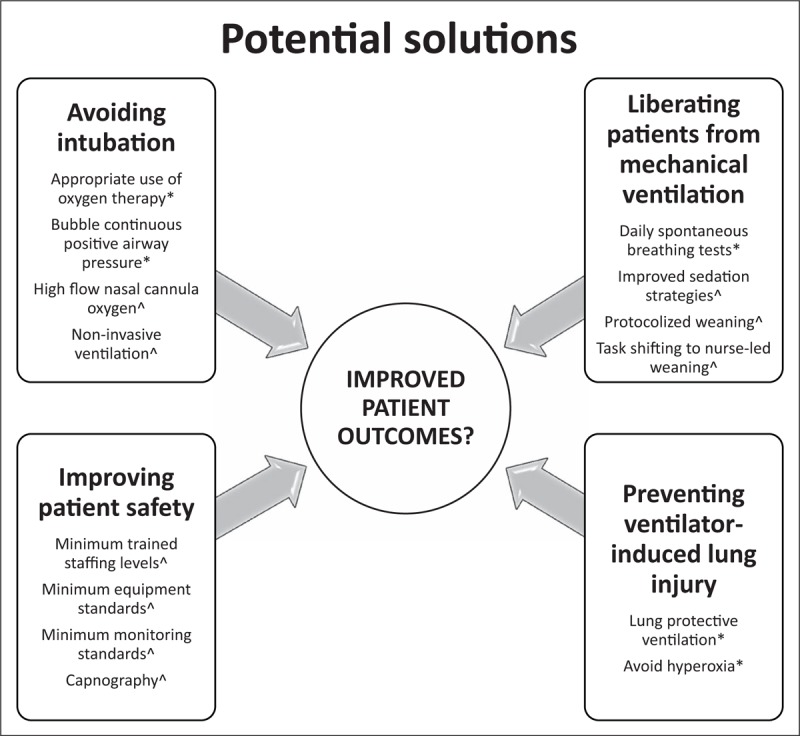
Proposed interventions to improve outcomes in patients with acute respiratory failure in low-income and middle-income countries. ^∗^Evidence-based interventions; ^^^Authors’ opinions only, with further research required.

In addition to avoiding mechanical ventilation where possible and expediting weaning, efforts should also focus on avoiding iatrogenic harm to the ventilated patient. Figure 1 summarizes how current interventions fit in with these overarching principles, distinguishing those interventions that have an established evidence base and those that are based on the authors’ recommendations only.

## EVIDENCE-BASED INTERVENTIONS

### Oxygen

The most fundamental treatment needed to support patients with acute respiratory failure is supplementary oxygen. It has been shown to reduce mortality in children with pneumonia by up to 35% and is on the WHO's list of essential medicines [30]. Nonetheless, a survey conducted among 97 clinicians from 19 countries across Africa, Asia and South America showed that only 32% of respondents reported working in a hospital with uninterrupted oxygen supplies [31].

Oxygen therapy should go hand in hand with pulse oximetry to target and monitor its use. Strategies to enhance institutional adoption of oximetry may be required [32^▪▪^]. It is worth noting, however, that there is no strong evidence to guide the threshold for giving oxygen, nor the optimal range of saturations to target in this, or indeed any, setting [33]. A large randomized trial is currently underway in Uganda and Kenya that seeks to answer this question in children. It is comparing liberal oxygenation, with target oxygen saturations of greater than 92%, to permissive hypoxemia, with saturations of greater than 80% [34]. As our appreciation of the risks of hyperoxia increases [33], combined with the need to optimize the use of a scarce resource, the need for clarity grows.

### Continuous positive airway pressure

One of the most significant developments in acute care research in LMICs in recent years has been the publication of three trials demonstrating that continuous positive airway pressure (CPAP) can reduce mortality in children under 5 years of age, compared with oxygen delivered via standard low-flow nasal cannula [35^▪^,^36^,37^▪^]. CPAP can also decrease the need for invasive mechanical ventilation [38^▪▪^]. There are three main ways to generate CPAP: first, by using a pressure driver or a ventilator; second, using high flow nasal-cannula oxygen therapy (HFNC); or third, by submerging the expiratory limb of a breathing circuit in water to create so-called bubble CPAP. Traditionally bubble CPAP circuits also contain a driver, although some newer iterations only use the oxygen/air flow from an oxygen concentrator to generate CPAP [39].

All three trials used bubble CPAP as the intervention and together showed a risk ratio of survival of 0.58 [95% confidence interval (CI) 0.41–0.82] [38^▪▪^]. One study had an additional intervention arm using HFNC, but no conclusions were drawn regarding its efficacy as the study was terminated early due to increased mortality in the control group.

Nasal cannulae, used as the patient interface in all three trials, are an attractive option for understaffed environments because they generally require lower levels of nursing supervision to use safely [39]. The basic circuits and simplified care protocols meant that the equipment required few adjustments, especially when compared with invasive mechanical ventilation.

There are elements of each of these studies that epitomize context-appropriate innovation and research. The bubble CPAP circuit deployed in the Bangladesh study was fashioned out of readily available, cheap equipment (standard nasal cannula, a shampoo bottle and intravenous fluid tubing) so the cost of the circuit was approximately $3 per patient [35^▪^]. They used an oxygen concentrator and no driver in the circuit with additional cost savings. The Malawi study team codesigned a bespoke, robust bubble CPAP device that cost $350 and required minimal maintenance [36]. The Ghanaian study promoted task-shifting by entrusting the initiation of respiratory support to nurses [37^▪^]. It was conducted in two district hospitals, outside the standard research setting of a tertiary hospital.

Nevertheless, the sustainability of new respiratory technology in a non-trial context is also a crucial consideration. 16 months after the close of a previous CPAP trial, the authors of the Ghanaian trial found that only 69% of the donated equipment was still both present and functional [40]. Furthermore, the CPAP system they implemented during the trial was dependent on single-use nasal cannulae that cost $32 per patient. These were imported and donated for the duration of the trial but were no longer being provided, leaving the host hospitals without access to this expensive consumable. The propagation of the skills needed to administer bubble-CPAP also proved inadequate; nurses trained by local staff on conclusion of the trial neither acquired nor retained all the necessary knowledge and skills.

With regard to adult practice in LMICs, the vast majority of CPAP therapy reported in the literature is delivered by ventilator [3^▪▪^]. Currently, there is no analogue to bubble CPAP for use in adults. However, HFNC oxygen therapy is the method of respiratory support that seems likely to prove most promising in this population. This technique requires minimal training to use safely. The nasal interface does not require an occlusive seal to generate CPAP and therefore needs less nursing oversight. It is generally well tolerated, sedation is unnecessary and it does not carry the risks of VAP.

We are not aware of any published trials looking at HFNC use in adult patients in LMICs, although the technology is currently in use in several middle-income countries. The major barrier to using HFNC in a low-resource environment is related to the very high oxygen requirements. A standard oxygen concentrator delivers oxygen flows of up to 10 l/min while HFNC in adults typically requires flows between 40 and 60 l/min. It would therefore require cylinder or piped oxygen and would be a heavy draw on a hospital's oxygen supplies. Even so, HFNC is an attractive target for future exploration.

### Noninvasive ventilation

In the most comprehensive review to date of the use of noninvasive ventilation (NIV) in LMICs, a 2018 systematic review and meta-analysis drew together data from 37 observational studies and 17 randomised controlled trials [3^▪▪^]. These included studies of bubble CPAP as well as ventilator-delivered NIV and covered both adult and pediatric populations. Importantly, it showed NIV to be a safe intervention in the settings in which the studies were conducted: mainly urban ICUs in Africa and South Asia, with 57% of the studies coming from India.

Despite the relatively low patient numbers, NIV was shown to reduce mortality in invasively ventilated adults, mostly with chronic obstructive pulmonary disease, who failed a spontaneous breathing trial versus ongoing mechanical ventilation. This mirrors findings in HICs and is helpful testament to the potential efficacy of NIV outside of a high-resource environment.

However, a key question when considering the potential role for NIV in LMICs is can it avert intubation in patients with hypoxemic respiratory failure? The findings here were more equivocal and require further exploration: 42% of adults with hypoxemic respiratory failure failed treatment with NIV (95% CI 33–51%; 15 studies, 461 patients) compared with 20% of adults with hypercapnic respiratory failure (95% CI 15–25%; 19 studies, 907 patients).

The biggest risk of NIV is the potential to delay a needed intubation, with the attendant mortality risk that can entail [41]. It also requires technical expertise and relatively close patient supervision to deliver effectively. Nonetheless, as NIV has been shown to be feasible and safe in certain LMICs, it remains a respiratory support modality that warrants further investigation.

### Invasive ventilation

The first set of recommendations to specifically address the respiratory management of mechanically ventilated patients in resource-limited settings was published in 2015 [42]. As the authors note, their literature search for studies of relevance from LMICs only turned up one randomized controlled trial and eight observational studies. As a result, many of the recommendations were extrapolated from evidence that originated in HICs. The key practice points are summarized in Table 2.

**Table 2:** Recommendations for ventilated patients in resource-limited settings

Recommendation	Grading
Elevate the head of the bed to 30–45°	1B
Use low tidal volumes of 5–7 ml/kg predicted body weight in ARDS patients and in all ventilated patients	1A/2B
Target oxygen saturations of 88–95%	2A
Use a minimum PEEP of 5 cmH_2_O	2B
Avoid high PEEP in patients who do not have an arterial line *in situ* as hypotension and circulatory depression may develop	2D
Use volume-controlled modes of ventilation in preference to pressure-controlled modes	2D
End-tidal CO_2_ monitoring could be helpful in timely recognition of over or under ventilation	2D
Use spontaneous breathing trials early and regularly, preferably daily	1A
When performing spontaneous breathing trials, use the low level of pressure support technique	2D
Only extubate patients when there are sufficient staff around to safely reintubate if needed	2D

ARDS, acute respiratory distress syndrome; PEEP, positive end-expiratory pressure. Adapted with permission from [42].

These recommendations address measures to decrease the risks associated with invasive mechanical ventilation and to promote liberation from the ventilator. Some are easier to follow in LMICs than others: for example, end-tidal carbon dioxide monitoring is not widely available due to prohibitive costs. A recent study in Malawi showed that there was just one capnograph in the entire country [43^▪^]. Notably, the same study demonstrated that it was both feasible and effective to introduce capnography into this setting, leading to early recognition of critical airway incidents.

Beyond these recommendations, we believe that there is minimum level of equipment, staff and training necessary to provide safe, quality care to the ventilated patient. In circumstances in which these minimum standards are not met, we would advocate against the use of mechanical ventilation at all. An example of one such situation would be an ICU without pulse oximetry or a functional suction device. The International Standards for a Safe Practice of Anesthesia adopt the same approach [44^▪▪^].

However, outside of these more extreme situations, there are non-evidence-based workarounds that may help alleviate some of the pressures on staff. Some examples encountered by the authors include task-shifting basic patient care tasks to families to free up nurses for more specialized roles; using time-saving devices such as closed suctioning; teaching simple physiotherapy interventions to the family; ensuring that the ventilator and monitoring alarms are set to appropriate limits so as to only trigger when an intervention is needed; and engaging the help of the family to keep the head of the bed elevated. These are in addition to clinical aide memoires such as intubation checklists or rounding pro formas, and the future prospect of clinical decision support [45,46^▪▪^].

The other aspect of supporting staff to provide quality care to ventilated patients is to address the psychological toll that working in such a challenging environment and caring for this high mortality group can take. Implementing measures to support staff can also help with motivation and retention; details of such measures are discussed elsewhere [2^▪▪^].

As for solutions to the equipment and infrastructure challenges, there are examples of companies that are producing resilient respiratory equipment designed to withstand many of the infrastructure difficulties encountered in low resource environments [47,48]. This superior level of design is essential to avoid contributing to the 70% of essential medical equipment in LMICs that is estimated to be nonfunctional [22^▪▪^]. Most notably, these companies ensure ongoing technical support and maintenance for the products they sell.

Another promising initiative is the African Biomedical Engineering Consortium, which links a network of African universities and aims to foster the skills required to develop robust and commercially viable medical devices [49^▪^]. The term frugal innovation has been coined to describe the design of an economical product that focuses on optimized performance and core functionality [50^▪▪^]. This approach has the potential to enable the provision of better respiratory support at a lower cost.

## CONCLUSION AND RECOMMENDATIONS

**FIGURE 2:**
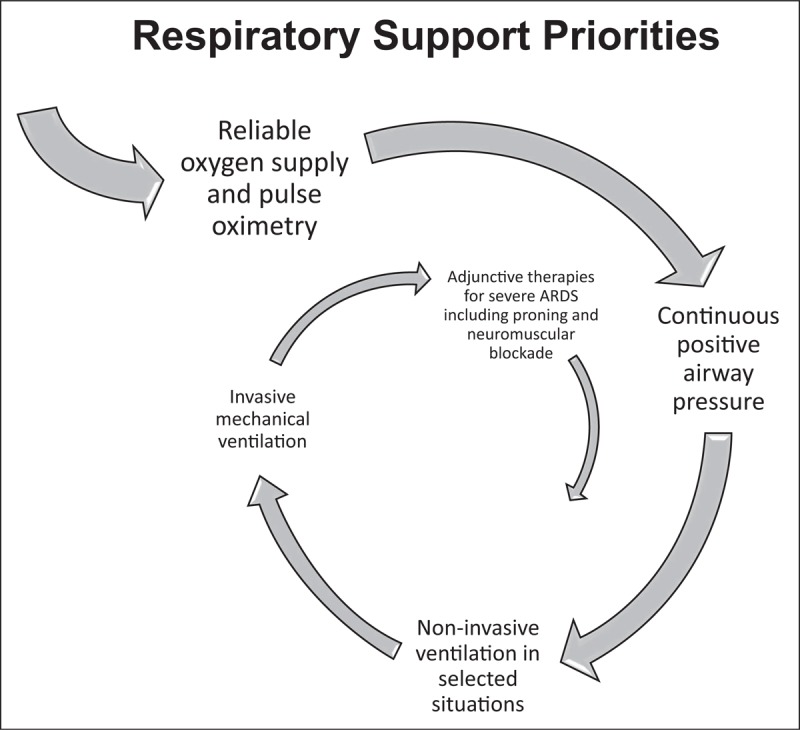
Proposed order of priority for respiratory support interventions.

Acute respiratory failure is a common, important problem worldwide and there is a definite need for more research into respiratory management in resource-limited settings. However, much of the excess mortality seen in ventilated patients in LMICs is likely to be due to incomplete application of what is already known, rather than to a dearth of research. To improve outcomes, attention needs to be focused on delivering basic critical care effectively rather than looking to rapidly scale up access to invasive mechanical ventilation (Fig. 2).

Furthermore, acknowledging the potential for significant iatrogenic harm in patients undergoing mechanical ventilation, it is important to prioritize straightforward measures to promote patient safety. We advocate for a minimum level of facilities and staffing to be available in an ICU prior to implementing mechanical ventilation services. These include continuous pulse oximetry while on the ventilator and the 24-h presence of a member of staff with sufficient airway training.

Finally, we appeal to industry and local innovators to emulate and expand the good practices being spearheaded by a small number of companies, embracing the principles of frugal innovation to tackle the challenges inherent to providing respiratory support in resource-limited ICUs.

The Lancet Commission has shone a spotlight on the 5 million deaths a year that could be avoided with high-quality healthcare [2^▪▪^]. Some of these deaths are evidently occurring in patients with acute respiratory failure. Now is the time for the critical care community around the world to champion a much-needed change.

## Acknowledgements

With thanks to Prof Arjen M. Dondorp for his support and advice.

### Financial support and sponsorship

None.

### Conflicts of interest

There are no conflicts of interest.

## REFERENCES AND RECOMMENDED READING

Papers of particular interest, published within the annual period of review, have been highlighted as:

▪ of special interest
▪▪ of outstanding interest
